# Synergistic enhancement of Al-Si7Mg alloy: Strengthening mechanical properties through combined electromagnetic agitation and AL-10%Ti refinement

**DOI:** 10.1371/journal.pone.0341127

**Published:** 2026-01-29

**Authors:** G. Shaikshavali, Din Bandhu, Rashi Tyagi, E. Venugopal Goud, Daniel Amoako Darko

**Affiliations:** 1 Department of Mechanical Engineering, G. Pulla Reddy Engineering College, Kurnool, Andhra Pradesh, India; 2 Department of Mechanical Engineering, Galgotias University, Greater Noida, Uttar Pradesh, India; 3 Institute for Environment and Sanitation Studies, College of Basic and Applied Sciences, University of Ghana, Legon - Accra, Ghana; Lovely Professional University, INDIA

## Abstract

Al-Si7Mg alloy is widely used in automotive and aerospace applications due to its favorable strength-to-weight ratio and corrosion resistance. However, further enhancement of its mechanical properties remains a key challenge. While grain refinement through chemical additives is common, the synergistic effect of combining a chemical grain refiner with external electromagnetic agitation during solidification has not been fully explored. This study investigates the simultaneous application of Al-10%Ti grain refiner and electromagnetic agitation, via a custom electromagnetic force (EMF) coil, on the microstructure and mechanical properties of Al-Si7Mg alloy. The EMF voltage was varied from 0 to 220 V. Microstructural analysis revealed that the combined treatment effectively transformed coarse, acicular silicon phases into a fine, closed-grain structure. This refinement is attributed to the electromagnetic agitation promoting uniform dispersion and increased potency of Al-10%Ti particles, which act as heterogeneous nucleation sites. The optimized microstructure led to significant improvements in mechanical properties: the sample treated with Al-10%Ti and 180 V EMF exhibited a maximum ultimate tensile strength of 173.3 N/mm², representing a 15% increase over the untreated baseline. Hardness improved by 21%, elongation increased by 60%, and wear resistance was notably enhanced, particularly at higher EMF levels (180–220 V). The results demonstrate that the combined approach offers a synergistic mechanism for microstructural refinement and property enhancement in Al-Si7Mg alloy, providing a promising route for advanced casting processes.

## 1. Introduction

High-integrity shaped aluminum castings have become increasingly vital to the automotive industry, primarily due to their superior corrosion resistance, excellent castability, and high strength-to-weight ratio. These properties are essential for meeting modern demands for vehicle weight reduction, which directly enhances fuel efficiency and reduces environmental emissions. Among these materials, Al–Si alloys are the industry standard, accounting for 85% to 90% of all aluminum cast components, due to their exceptional mechanical properties and manufacturing versatility [1–4]. Al-Si7Mg (LM 25) alloy is extensively utilized in critical automotive and aerospace components where high strength-to-weight ratio, fatigue resistance, and durability are paramount. In the automotive sector, it is a preferred material for engine blocks, cylinder heads, and transmission casings, which are subjected to severe cyclic mechanical loading, thermal stresses, and wear. The improvements in ultimate tensile strength (∼15%) and hardness (∼21%) demonstrated in this study directly enhance the load-bearing capacity and resistance to deformation of these components, potentially leading to more compact, efficient engine designs. Similarly, the significant increase in elongation (∼60%) indicates improved toughness and damage tolerance, crucial for absorbing impact energy and resisting crack initiation under cyclic loads. In aerospace applications, such as for pump bodies, brackets, and non-critical structural frames, these enhanced mechanical properties contribute to greater structural integrity and reliability while maintaining essential weight savings.

Furthermore, the markedly improved wear resistance resulting from the synergistic grain refinement is particularly beneficial for moving parts and surfaces in contact, reducing maintenance needs and extending service life. Therefore, the property enhancements achieved through combined electromagnetic agitation and Al-10%Ti refinement have a direct and substantial influence on the alloy’s usability, performance, and safety in these demanding applications [5–8]. The alloy’s components and microstructure are crucial to achieving the optimal mechanical properties. One of the first alloy systems is aluminium-silicon, which is mostly utilized for castings. Silicon contributes wear resistance, low shrinkage, and fluidity. Silicon, however, reduces its toughness. Mechanical Properties of Aluminium are determined by the composition of the alloy and its innate elemental qualities [9–11]. The quality of the cast can be improved by adding elements to the melt during alloy melting, subjecting the alloy to a prolonged heat treatment, subjecting the melt to mechanical vibrations during solidification, and subjecting the alloy to plastic deformation.

Grain refining can be accomplished using rapid solidification [12]. To achieve a finely equiaxed grain structure in Al-Si alloys, grain refining is crucial. An equiaxed grain structure ensures consistent mechanical properties, reduces ingot cracking, improves feeding to eliminate shrinkage porosity, provides a fine-scale distribution of second phases and microporosity, and enhances casting machinability [13]. Research on the Al-10%Si-35%Mg alloy with Al-1.5%Ti-1.5%B and Sr added revealed that the Al-Ti-B master alloy had a positive refining effect, and the impact of Sr addition was minimal [14]. The addition of the refiner to the melt alters long, extended α´-Al grains into fine, equiaxed α´-Al grains. Both nucleation and growth lead to the production of free equiaxed crystals. The existence of strong nucleating particles does not ensure a finely grained outcome. Primary crystallization is the term used to describe the transition of a metal from a liquid to a solid state. At this point, the molten metal begins to solidify into grains, with the molten metal surrounding the nuclei. The latent heat collected during melting is continuously released by the nuclei and the molten metal hardening around them [15]. Dendritic formation occurs when the temperature drops as a result of cooling, causing the nuclei to expand quickly and atoms to adhere to one another in similar layers around the nuclei. Nucleation occurs through two mechanisms: Homogeneous nucleation and Heterogeneous nucleation. In homogeneous nucleation, the stability of a nucleus is controlled by two factors: the free energy change during the liquid phase and the value of the energy of the nucleus thus formed [16].

Mechanical agitation, when applied to metals and alloys during the solidification process, alters the macro- and microstructures that were previously formed through mechanical vibrations at both low and high frequencies. The creation of a fine-grained equiaxed structure and the suppression of undesired dendritic and columnar zones are the most frequently seen effects. During solidification, the dendritic points continue to grow and solidify, eventually impinging on one another to form dendritic grains. This point is referred to as the dendrite coherency point. Here, the coherency fraction, coherency temperature, and coherency time refer to the fraction solid, temperature, and solidification time, respectively [17]. The directional solidification technique aids in crystal growth in aluminium-copper eutectic and hypoeutectic alloys. There is a prevalence of columnar zones that have significant temperature gradients and small growth velocities. It is possible to freeze columnar grains in a specific orientation, resulting in a specific crystallographic direction on the long axis. In contrast, Certain grains of equiaxed dendrites are oriented randomly [18]. Due to their exceptional combination of low weight, strength, corrosion resistance, and formability, aluminium alloys are vital in a wide range of sectors. Aluminium alloys, such as 2024, 6061, and 7075, as well as aluminium-lithium, are used extensively in the aerospace industry for the construction of aircraft structures, wings, and fuselage components. These alloys provide components with an ideal weight-to-strength ratio and good fatigue resistance. As a result, cargo capacities are increased, fuel economy is improved, and operations are made safer. Similarly, aluminium alloys are widely used in the automobile industry for the production of engine blocks, wheels, body panels, and suspension management systems [19]. Aluminium alloys are used in the building sector for various structural purposes, including doors, windows, facades, roofs, and staircase railings. Aluminium alloys of series 3000 and 5000, renowned for their simplicity of production and resistance to corrosion, are particularly popular in architectural projects that seek to achieve both durability and aesthetic appeal. Additionally, due to their ability to withstand adverse climatic conditions, these alloys are preferred for use in the construction of bridges and buildings along the coast [20]. Their high electrical conductivity enables them to be used in power transmission, household wiring, and electronic packaging, all of which are important aspects of energy management. Aluminium alloys also play a crucial role in energy management [21]. Aluminium alloys are suitable for use in the packaging industry due to their impermeable and non-toxic properties, making them suitable for use in food and pharmaceutical containers. Additionally, its high reflectivity is utilized in the production of mobile phones, as well as in the creation of mirrors and insulation [22]. The extensive usage of aluminium alloys in the aerospace, automotive, construction, energy, electrical, packaging, and consumer goods sectors is driven by the adaptability and customizable qualities of aluminium alloys. Not only does the combination of their low weight, strength, resistance to corrosion, and processability contribute to increased engineering efficiency, but it also provides considerable advantages to the environment and the economy [23]. A fine, coaxial grain structure is the objective of aluminium billet casting. Grain type and billet size depend on chemical makeup, grain refiners, as well as the speed of solidification. The casting process is made more efficient by grain refiners, as they reduce the occurrence of self-shrinkage, hot tearing, and hydrogen porosity. Mechanical qualities are improved in products that are produced by the extrusion of billets that have been poured through the grain refining process. Heat treatment has a greater impact on its sensitivity. In addition to being more resistant to surface ripping, they are also capable of producing superior surfaces as a consequence of chemical and electrochemical surface treatments [24]. According to the findings, the AlTi5B1 master alloy, prepared in rod shape and containing efficient nucleating elements such as boron and titanium, was the preferred choice for use in the casting process as a grain refiner. Grinders in Al-Si alloys are primarily responsible for the regulated development of grain structures via heterogeneous nucleation. This is the major function of grain refiners [25]. Titanium is generated by the formation of the compound TiAl3 in the structure, which causes heterogeneous nucleation during the process, as stated by the grain reduction hypothesis. A heterogeneous nucleation mechanism is responsible for the nucleation of aluminium on the TiAl3 compound during the cooling process. Moreover, its fine-grained structure is produced by its own mechanisms. This occurs without the need for any overcooling to take place. Homogeneous nucleation occurs within a homogeneous liquid when it solidifies in the absence of nucleating particles, resulting in the formation of a solid phase. Significant driving power is required for homogeneous nucleation, necessitating that the nucleation process commences at a greater undercooling (at a temperature lower than the equilibrium melting point). Conversely, the presence of powerful nucleant particles inside the melt induces heterogeneous nucleation, which requires a reduced driving force and initiates at a lower degree of undercooling. The transition from liquid to solid is contingent upon the removal of heat from the liquid phase. It is well recognized that an elevated cooling rate during solidification results in a microstructure characterized by finer grains [26]. It is possible to quickly lower the solidification temperature of die castings, permanent metal mold castings, and thin-walled castings. Mechanical vibration therapy is recognized for its ability to produce microstructural refinement. Nonetheless, the specific element of vibrations critical for microstructure refinement remains inadequately defined. Factors influencing vibrations encompass amplitude, velocity, acceleration, and frequency. Our investigation revealed that the rates at which mechanical vibrations occur, also known as the power of vibrations, are a crucial component for the refining of primary crystals [27]. The introduction of mechanical vibration causes coarse dendritic structures to transform into fine particles that are evenly matched during the process. The size, form, and distribution of the eutectic silicon particles and the α-Al primary phase exhibited significant differences; in addition to the SDAS, considerable enhancements have been observed. Furthermore, the degree of grain growth has grown as the wall thickness has increased. The relative motion of the liquid metal, either before (while it is still within the crucible) or after (while it is still inside the mold), pouring has the potential to precipitate grain refining. The impurities, which include oxides, intermetallics, and nonmetallic compounds, are evenly disseminated when the melted alloy is subjected to agitation and pressure to flow prior to or during the hardening process. Additionally, the hardened dendritic points fracture and migrate into the liquid metal. There are more possible heterogeneous nucleation sites when there are more solid particles within the melt. The primary cause of grain refining by agitation is the increased quantity of nucleants present in the liquid and the uniform dispersion of these particles [28]. To achieve dispersive mixing, intense shearing is required, whereas for distributive mixing, a macro-flow is created within a volume of melt. Additionally, the high shear device demonstrates significantly improved kinetics for phase transformations, uniform dispersion, distribution, and size reduction of solid particles and gas bubbles, as well as enhanced homogenization of chemical composition and temperature fields. Furthermore, it facilitates forced wetting of solid particles that are typically difficult to wet, such as those in liquid metal. Solid particles, either introduced to or generated in the molten metal itself, are known as grain refiners. Grain refiners are often incorporated into the melted metal as fundamental alloys. Fundamental principles exist in order for a solid particle to perform the job of a grain refiner and to assist in the nucleation of a metallic alloy. The element units must remain intact and unaffected by the melt’s constituents in order for it to be considered stable [29]. There ought to be a slight crystallographic mismatch between the particles and the phase of nucleation. A minor wetting angle is desirable between the particle and the developing solid [30]. It’s also crucial to consider the disparity in density between the particle and the molten metal. The particle must remain intact and unaffected by the melt’s constituents in order for it to be considered stable.

A little crystallographic mismatch is required between the nucleating phase and the particle [31]. The contact particle’s angle with respect to the solidifying material should be minimal [32]. There is a large density difference between the particle and the metal in a liquid state. Suppose the particle’s weight is too high or too low relative to the melt. In that case, it will result in particles settling or floating, causing the grain refiner to be distributed unevenly throughout the melt. During the solidification process, the alloy’s nucleation sites are solid particles, which in turn increase the total quantity of nucleated grains within the substance.

Another method of improving Al-Si7Mg alloy properties is by reinforcing it with hybrid particulates of silicon carbide (SiC), graphite (Gr), and zirconia (ZrO₂) at varying weight fractions of 3, 6, and 9 wt% to improve mechanical and corrosion characteristics. The composite with the lowest reinforcement fraction exhibited superior hardness, compact microstructure, and the best resistance to corrosion in saline conditions. Moderate reinforcement improved impact strength, while excessive addition reduced flexural strength due to matrix embrittlement. Electrochemical and microstructural analyses revealed lower corrosion activity in the optimally reinforced composite, with surface examinations confirming pitting as the dominant corrosion mechanism [7].

## 2. Materials and methods

The alloys chosen for the study are the Al-Si7Mg alloy (LM 25). Baird’s spectrometer was used to perform spectral analysis on the alloys to determine their composition, and the results are presented in Table 1.

**Table 1:** Chemical composition of Al-Si7Mg (LM25) alloy.

Element	Cu	Mg	Si	Fe	Mn	Ni	Zn	Pb	Sn	Ti	Al
Max	0.2	0.6	7.5	0.5	0.3	0.1	0.1	0.1	0.05	0.2	Bal

**Fig 1:**
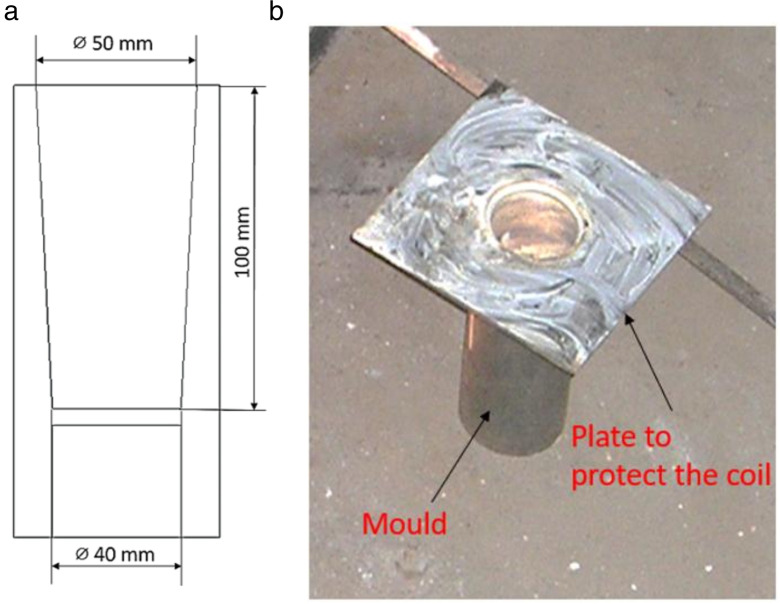
(a) Schematic diagram of the permanent steel mould design welded with a metal plate for electromagnetic agitation; (b) Photograph of the actual EN19 steel mould used for casting the Al-Si7Mg alloy samples. Fig 15. Variation of wear rate versus load for various voltages applied with refiner addition.

The material was obtained from a local manufacturer, produced in the form of ingots. The refiner chosen for grain refinement is AL-10%Ti. The amount of Al-10%Ti master alloy to be added to achieve a titanium addition level of 0.05% in the Al-Si7Mg alloy melt is calculated as follows. The target level of the grain element addition rate is 0.05% Ti. The quantity of Al-Si7Mg melted is 5 kg. The weight of titanium required is 0.05% × quantity of Al-Si7Mg in grams, which amounts to 0.05 × 2.5 grams, or 0.125 grams. The percentage of titanium present in AL-10%Ti master alloy is 10%. Therefore, the weight of the AL-10%Ti master alloy to be added is 25 grams. A permanent mold is used for casting the samples. The mould is made of EN19 steel. The mold is designed to cast circular castings measuring 100 mm in length and 40 mm in diameter (Fig 1). The EN19 plate was welded to the permanent mold for ease of removal and insertion into the EMF coil.

Mechanical agitation of the melt was ensured by inducing mechanical vibrations with the help of a coil that produces an electromagnetic force effect (EMF) in the steel mould. The intensity of the emf varies by altering the voltage input of the coil. Details of the coil used are given in Table 2.

**Table 2:** Specifications of coil assembly.

Metal used	copper
No of turns	1500
Wire gauge	14 gauge
Shape	circular
Height	135 mm
Internal diameter	65 mm
External diameter	80 mm

**Fig 2:**
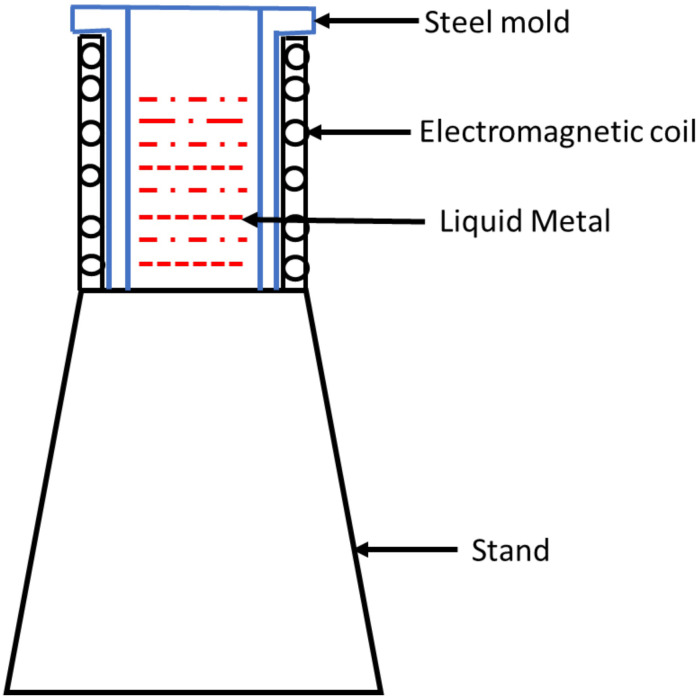
Nomenclature of EMF coil assembly.

The windings were wound on a core with a diameter of 64 mm, which had a thermal lining insulation coating. Thermal insulation was applied over these windings of coils to guard the windings from high temperature (Fig 2). A thick coating of epoxy substance was applied to this, making it watertight. To create the electric circuit and change the electromotive force (emf), several taps were supplied. An electrical connection was established for the input.

**Fig 3:**
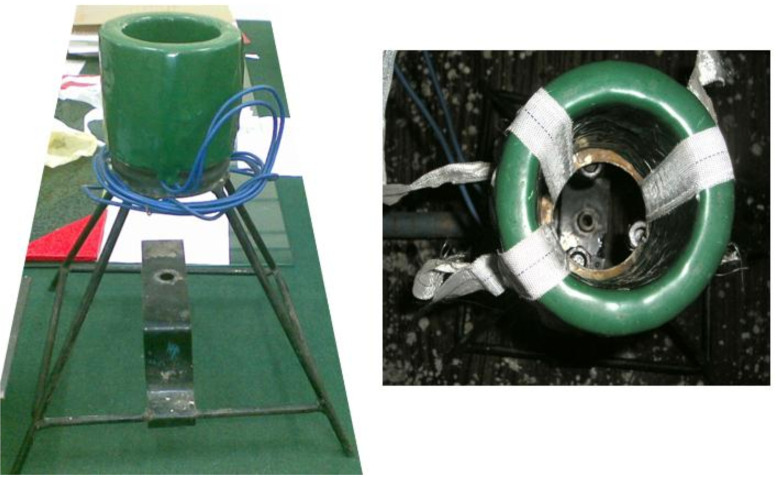
EMF coil with a stand.

The magnetic field can then be varied by adjusting the voltage using the autotransformer. The voltage was varied from 50 volts, 100 volts, and 120–220 Volts in substeps of 20 volts. Fig 3 shows the coil assembly with a stand.

**Fig 4:**
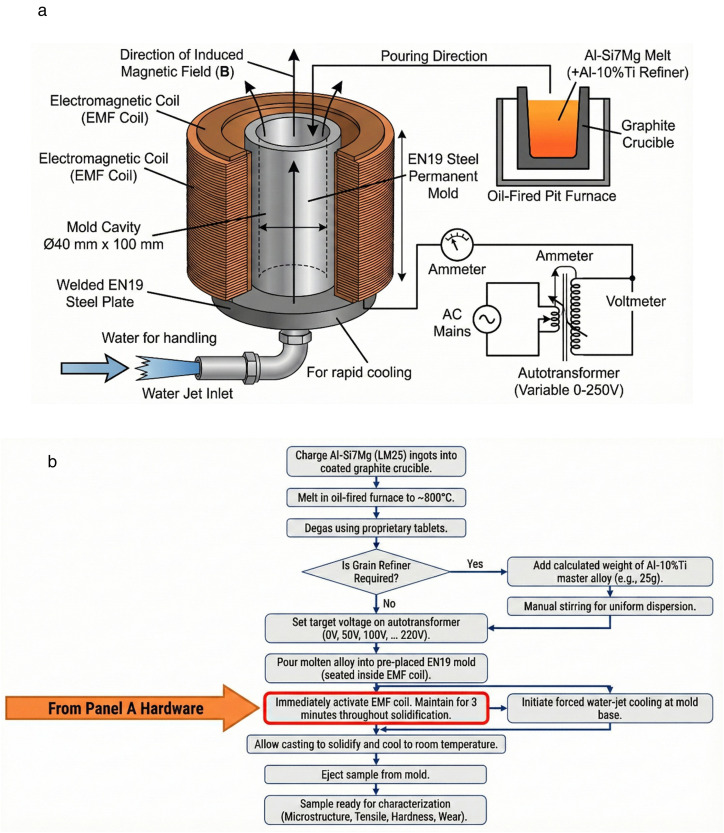
Schematic of the electromagnetic agitation setup and stepwise solidification process for Al-Si7Mg alloy treatment. **(a)** Hardware Setup Schematic (Panel A). **(b)** Stepwise Process Flowchart.

Fig 4 details the schematic of the electromagnetic agitation setup and solidification process used in the present investigation. The unit (Fig 4a) consists of a pod stand to provide provision for seating the mould and coil assembly in the proper position, and a water jet assembly to rapidly cool the mould. The coil setup was connected to the electrical assembly, which consisted of an ammeter, voltmeter, and autotransformer to vary the magnetic field. A Chromel-Alumel thermocouple, along with a digital temperature indicator, is used to measure the temperature of the melt. Fig 4b depicts the corresponding stepwise experimental procedure. The EMF was applied immediately after pouring and maintained throughout solidification under concurrent water-jet cooling.

**Fig 5:**
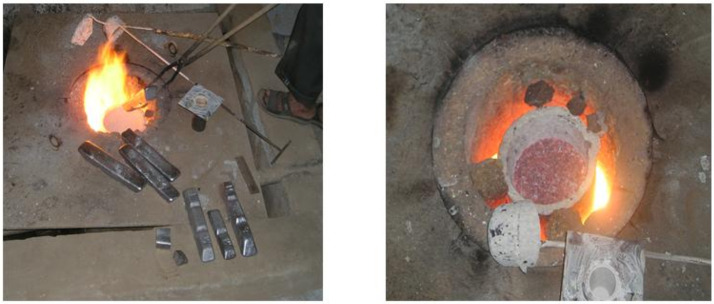
Oil-fired diesel furnace of the pit type for alloy melting.

An oil-fired diesel furnace of the pit type was used to melt the alloy, as shown in Fig 5. After coating the graphite crucible with fresh lime, it was placed in the pit furnace, and the Al-Si7Mg alloy in ingot shape was transferred into the crucible. The furnace is fired up to melt the alloy. The metal is heated to temperatures of 800 °C after attaining the melt temperature, and degassing is carried out using proprietary degassing tablets.

The samples were cast as (a) without the inclusion of a refiner and excluding the induction of a magnetic field. (b) Inducing the magnetic field into the melt without a refiner. (c) By adding a refiner without a magnetic field. (d) By adding a refiner with a magnetic field. For rapid cooling, cold water is continuously forced to impinge at the bottom of the mould. A magnetic field was induced into the melt for 3 minutes until the metal solidified. The concentration of the magnetic field was changed by increasing the voltage, with variations used being (50V, 100V, 120V, 220V) in steps of 20 volts. After solidification and allowing the samples to reach room temperature, they were expelled from the mold. Table 3 shows the details of samples cast with grain refiners and varying (EMF) to the melt

**Table 3:** Details of samples cast with grain refiners and varying (EMF) to the melt.

Sample No.	Particulars EMF applied for 3 min	Volts
1	Without refiner, without EMF(As cast)	0
2	Without refiner, without EMF after degasser	0
3	Without refiners, with (EMF)	50
4	Without refiners, with (EMF)	100
5	Without refiners, with (EMF)	120
6	Without refiners, with (EMF)	140
7	Without refiners, with (EMF)	160
8	Without refiners, with (EMF)	180
9	Without refiners, with (EMF)	200
10	Without refiners, with (EMF)	220
11	With refiner, without EMF	0
12	With refiners and magnetic field (EMF)	50
13	With refiners and magnetic field (EMF)	100
14	With refiners and magnetic field (EMF)	120
15	With refiners and magnetic field (EMF)	140
16	With refiners and magnetic field (EMF)	160
17	With refiners and magnetic field (EMF)	180
18	With refiners and magnetic field (EMF)	200
19	With refiners and magnetic field (EMF)	220

Several key assumptions were considered during the preparation and extraction of samples across all EMF conditions to ensure procedural consistency and the validity of comparative analysis. First, it was assumed that the Al-10%Ti grain refiner was added to the melt only after the Al-Si7Mg charge had reached a complete and homogeneous molten state, ensuring proper dissolution and interaction. Second, sufficient holding time was allowed after refiner addition for complete melting and uniform mixing within the melt. Third, to augment this mixing prior to EMF application, the melt was stirred manually using a graphite stirrer, establishing a consistent initial distribution of nucleants. Fourth, the electromagnetic field was applied immediately after pouring and maintained continuously for a duration of three minutes, covering the critical period until complete solidification of the melt within the mold. Finally, all cast samples were assumed to undergo similar cooling conditions after solidification; they were extracted from the steel mold and allowed to cool uniformly in ambient air. These controlled steps were fundamental in isolating the effects of EMF voltage and grain refiner from variations in processing parameters [33].

### 2.1 Microstructure examination

For the microanalysis, an abrasive wheel cutter was used to cut the material. An abrasive belt grinder was used for rough grinding to eliminate sharp edges, burns, and distorted material. Abrasive papers with silicon carbide grits of 100, 220, 400, and 600 were used in stages for the grinding process. Silicon carbide sheets with grits of 800 and 1000 are used for fine grinding. Final polishing is completed using a micro-polishing cloth, along with fine polishing diamond paste. After polishing, samples are cleaned using distilled water and dried thoroughly. Samples were made visible by etching with Keller’s reagent, which reveals the grain structure. With the following composition: HCl (1.5 mL), HNO3 (2.5 mL), HF (1.0 mL), and H2O (95 mL), the samples were cleaned and dried thoroughly after etching. After that, the etched samples were examined at several magnifications using an optical microscope.

### 2.2 Tensile test

**Fig 6:**
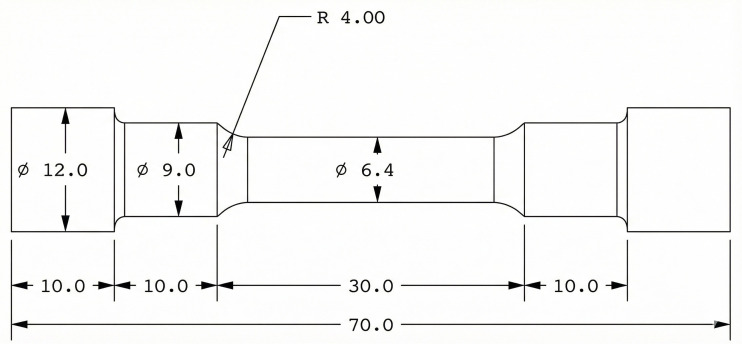
Tensile test specimen.

Tensile tests were conducted using a horizontal tensile tester, and specimens were prepared according to the ASTM E8 standard, which is commonly used in the aerospace and automotive industries, as shown in Fig 6. The tests were conducted for all specimens, both without and with the addition of refiner, at different EMF conditions.

### 2.3 Hardness test

**Fig 7:**
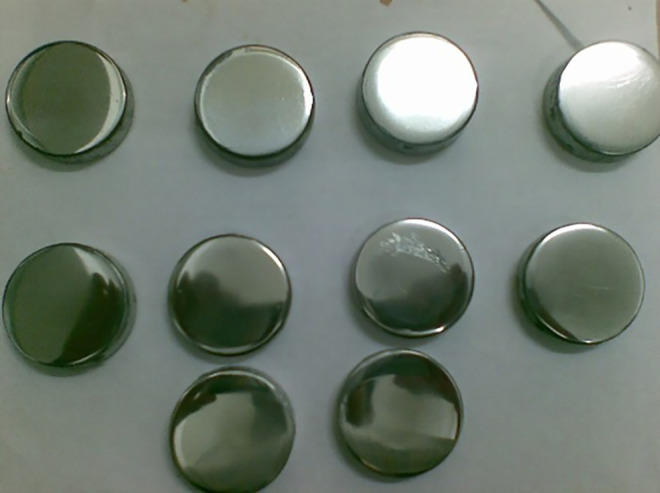
Hardness test samples.

Brinell hardness testers were used to perform a hardness test (Fig 7). The Standard procedure was followed, with measurements taken at four locations in the sample, and the average reading was considered for the study.

## 3. Result and discussion

### 3.1 Microstructure analysis

**Fig 8:**
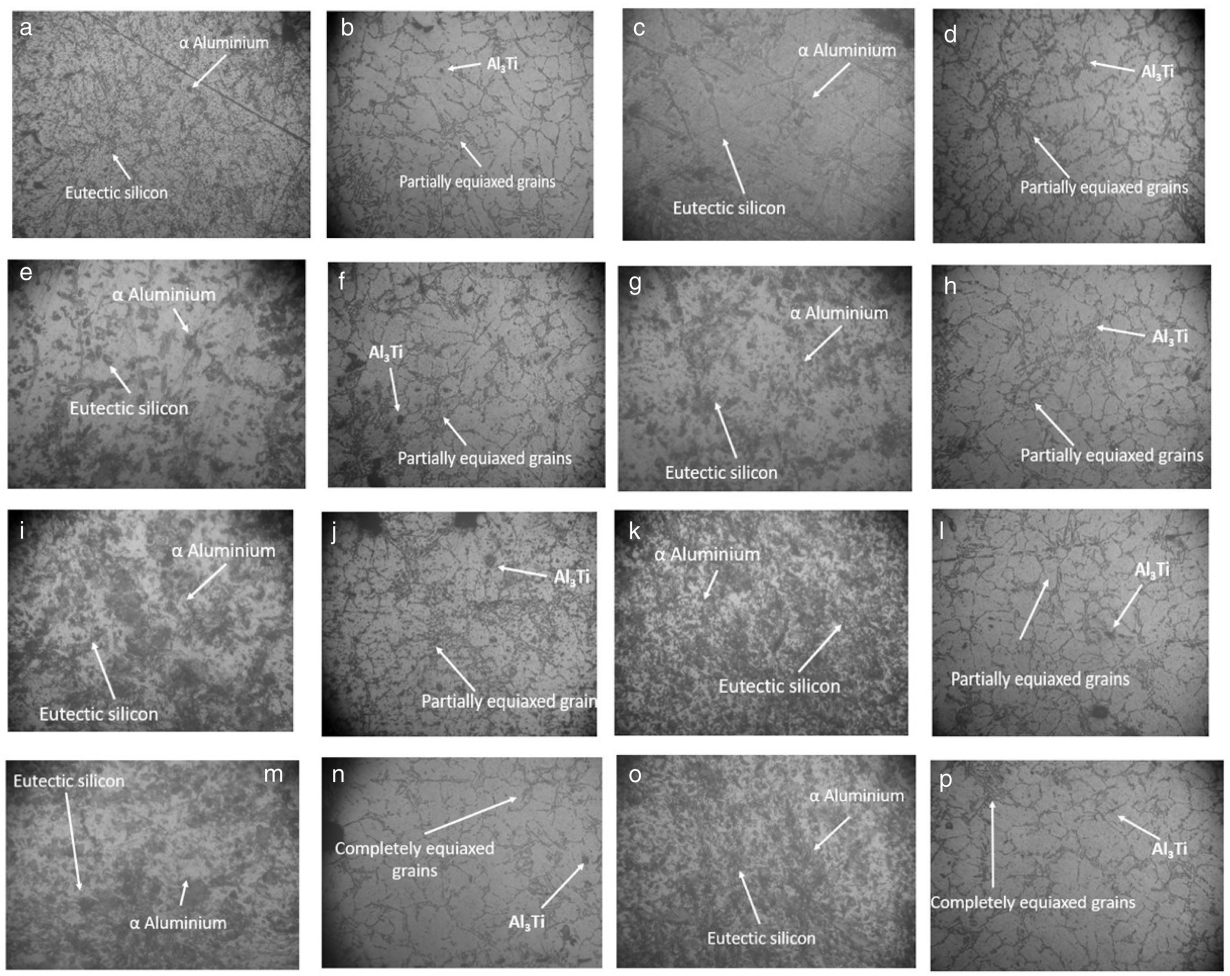
The optical micrograph of the specimens that are subjected to an electromagnetic field (EMF) at 220V. It is evident that the size of the grains has decreased, and a close grain is visible in the image. Al-Si-alloy microstructures are made up of an aluminium matrix reinforced by Si precipitates, as well as a mixture of Fe-rich intermetallic and eutectic silicon particles. Micrograph of specimens subjected to an electromagnetic field (EMF) at 50V shows that, with the use of a refiner (AL-10%Ti), the grain size is reduced and a close grain structure is observed. **(a)** sample subjected to EMF at 50Volts. **(b)** with refiner (Al-10%Ti) subjected to EMF at 50Volts. The optical micrograph of the specimens that are subjected to an electromagnetic field (EMF) at 100V, the size of the grain is decreased, and the grain is noted to be close together. It is evident from the optical micrograph of the specimens exposed to an electromagnetic field (EMF) at 100 volts that the refiner (AL-10%Ti) reduces grain size and produces a close-grained structure. **(c)** subjected to EMF at 100Volts. **(d)** with refiner (Al-10%Ti) subjected to EMF at 100Volts. Optical micrograph of specimens subjected to an electromagnetic field (EMF) at 120V, with a refiner (AL-10%Ti). The grain size decreases, and close grains can be observed. **(e)** subjected to EMF at 120Volts. **(f)** with refiner (Al-10%Ti) subjected to EMF at 120Volts. The optical micrograph of specimens subjected to an electromagnetic field (EMF) at 140V shows that, with the refiner (AL-10%Ti), the grain size is reduced and a close grain structure is observed. **(g)** subjected to EMF at 140Volts. **(h)** with refiner (Al-10%Ti) subjected to EMF at 140Volts. The optical micrograph of specimens subjected to an electromagnetic field (EMF) at 160V shows that, with the refiner (AL-10%Ti), the size of the grains is decreased, and a close grain structure is visible in the image. **(i)** subjected to EMF at 160Volts. **(j)** with refiner (Al-10%Ti) subjected to EMF at 160Volts. The optical micrograph of the specimens that are subjected to an electromagnetic field (EMF) at 180V. It can be observed that there is a decrease in grain size and a tight grain structure. **(k)** subjected to EMF at 180Volts. **(l)** with refiner (Al-10%Ti) subjected to EMF at 180Volts. The optical micrograph of the specimens that are subjected to an electromagnetic field (EMF) at 200V. There is a decrease in grain size, accompanied by a tight grain structure. **(m)** subjected to EMF at 200Volts. **(n)** with refiner (Al-10%Ti) subjected to EMF at 200Volts. **(o)** subjected to EMF at 220Volts. **(p)** with refiner (Al-10%Ti) subjected to EMF at 220Volts.

Microstructure examination was carried out using an optical microscope. The microstructure of the as-cast sample is shown in Fig 8(a)–Fig 8(p), with Si particles scattered throughout its boundary.

The addition of the Al–10% Ti master alloy to the molten alloy causes the decomposition of compounds of Al3Ti phase platelets, which then get engulfed inside the a-Al dendrites.

As the primary Al3Ti phase begins to precipitate, the permissible limit to the quantity that can participate in the enhanced nucleation of the main aluminium stage during solidification, which occurs in the peritectic reaction, is reached. This reaction is responsible for improving the solidification process.

### 3.2 Mechanical properties characterization

**Fig 9:**
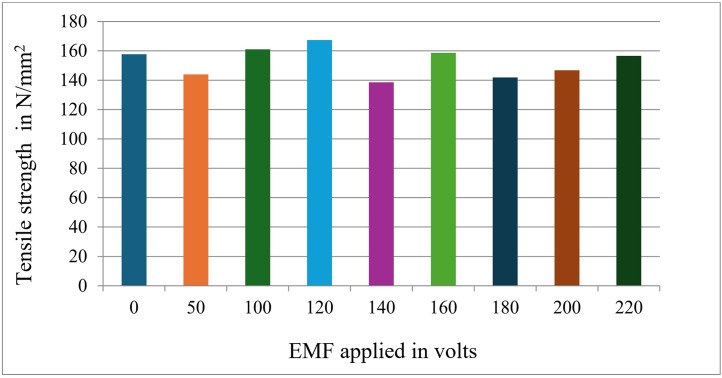
Variation of ultimate tensile strength (UTS) with applied electromagnetic field (EMF) voltage for Al-Si7Mg alloy without grain refiner. The peak at ~120V indicates an optimal agitation intensity for grain refinement, beyond which strength decreases due to increased defect formation.

Fig 9 displays the details of the UTS values of specimens subjected to different EMF conditions. It is observed that the tensile strength value increases with an increase in the electromagnetic effect.

The trend observed in Fig 9, where ultimate tensile strength (UTS) increases to an optimum at approximately 120V EMF before declining at higher voltages (up to 200V), can be explained by the competing effects of electromagnetic agitation on solidification dynamics. The initial increase in UTS is attributed to progressive grain refinement induced by the electromagnetic field. The Lorenz forces generated by the EMF create intense forced convection within the molten alloy [34,35]. This convection enhances thermal and compositional homogeneity, reduces the temperature gradient at the solidification front, and, most critically, fragments the developing dendritic arms. These fragmented dendrites act as additional nucleation sites, promoting a transition towards a finer, more equiaxed grain structure, a well-documented mechanism known as dendrite fragmentation or ‘crystal multiplication’ [22,36]. The consequent grain refinement strengthens the material via the Hall-Petch relationship.

However, beyond an optimal agitation intensity (here, ~ 120V), the beneficial effects are counteracted by detrimental phenomena. Excessive electromagnetic stirring can lead to elevated turbulence, which increases the entrapment of oxide bifilms and gas porosity within the solidifying structure [37, 38]. Furthermore, very high fluid flow velocities can cause remelting of critically sized nuclei or prevent the stable growth of equiaxed grains, ultimately coarsening the microstructure or introducing casting defects. This non-monotonic relationship between agitation intensity and mechanical properties, where an optimum exists, aligns with findings in similar systems employing intense melt shearing or ultrasonic processing [39,40]. Therefore, for Al-Si7Mg alloy without a grain refiner, 120V represents the threshold where the grain-refining benefits of EMF are maximized before defect generation dominates at higher voltages.

#### 3.2.1 Tensile stress for AL-Si7Mg with (AL-10%Ti) refiner.

**Fig 10:**
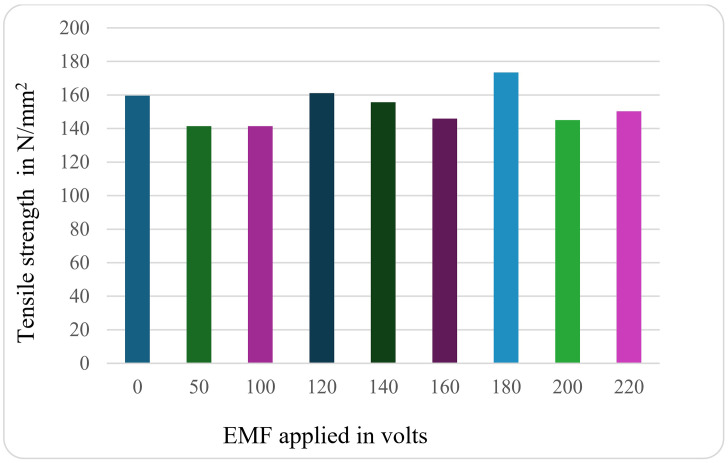
Variation of ultimate tensile strength (UTS) with applied electromagnetic field (EMF) voltage for Al-Si7Mg alloy with Al-10%Ti grain refiner. The sustained increase up to 220V demonstrates the synergistic effect, where EMF agitation optimizes the distribution and potency of refiner particles, leading to progressive grain refinement.

Fig 10 shows the details of the tensile values of specimens subjected to different EMF conditions for the Al-Si7Mg alloy with (AL-10%Ti) refiner. It is seen that the tensile strength value increases with high electromagnetic effect. The maximum tensile stress value was observed for the voltages of 120V, 180V, and 220V, which are subjected to an electromagnetic effect.

The sustained enhancement of ultimate tensile strength (UTS) with increasing EMF voltage up to 220V for the Al-10%Ti-refined alloy, as shown in Fig 10, demonstrates the critical synergy between chemical inoculation and electromagnetic agitation. In this system, the Al-10%Ti master alloy introduces a high population of potent, heterogeneous nucleation sites, primarily in the form of TiAl₃ particles [32,41]. The primary function of the electromagnetic field is to activate and optimize the efficiency of these nucleants. The forced convection generated by the EMF performs two key functions: first, it ensures the uniform spatial distribution of TiAl₃ particles throughout the melt volume, preventing their gravitational settling or agglomeration [42]; second, it continuously fragments the developing solidification front, creating a high number of free-floating dendritic fragments that further seed equiaxed growth [22].

This combined action results in a dramatic increase in the effective number of nucleation events. Consequently, a very fine, fully equiaxed grain structure is achieved, whose strengthening is accurately described by the Hall-Petch relationship [43]. Unlike the system without a refiner (Fig 9), where excessive agitation beyond an optimum (~120V) introduces defects that degrade properties, the presence of abundant, well-dispersed inoculant particles provides a stable substrate for nucleation even under intense fluid flow. The agitation, rather than becoming detrimental, becomes increasingly effective at distributing nuclei and fragmenting grains as the voltage rises. The performance peaks observed at 120V, 180V, and 220V in Fig 10 likely correspond to successive thresholds where the EMF intensity becomes sufficient to fully disperse and then optimally utilize the available nucleant population, culminating in the maximum UTS of 173.3 N/mm² at 180V. This synergistic mechanism, where external fields enhance the efficiency of grain refiners, is supported by analogous findings in systems using ultrasonic treatment with Al-Ti-B or Al-Nb-B refiners [44,45].

The tensile strength of Al-Si7Mg alloy increased mainly because of grain refinement through refiner AL-10%Ti and formation of hard, stable intermetallic particles that hinder dislocation motion. The Ti from Al--10%Ti forms intermetallics such as Al₃Ti that act as potent nucleation sites for primary α-Al grains. The Hall-Petch effect improves strength because more nuclei result in numerous smaller, equiaxed grains. This microstructural origin for the enhancement of tensile strength is strongly supported by recent work on a similar Al-7Si system, where ultrasonic agitation coupled with a grain refiner (Al-5Nb-1B) was shown to produce a refined globular microstructure directly leading to superior ultimate tensile strength, corroborating the efficacy of combined external energy and inoculant strategies [44].

#### 3.2.2 Effect of EMF on AL-SI7Mg alloy with and without the addition of refiner.

**Fig 11:**
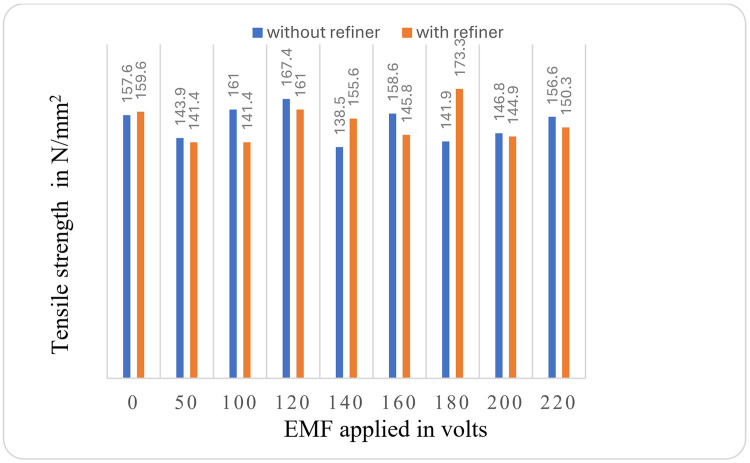
Comparison of Tensile strength values for Al-Si7Mg alloy with and without the addition of refiner.

The comparison of tensile strength of specimens (Fig 11) shows the effect of EMF on casted samples with and without grain refiner, there is a increase in tensile strength at the 120V EMF effect for both with and without addition of grain refiner, further the tensile strength of the sample with refiner at 180V EMF effect shows the tensile strength of 173.3 N/mm^2^ which is the maximum of all the samples tested.

#### 3.2.3 Hardness evaluation.

**Fig 12:**
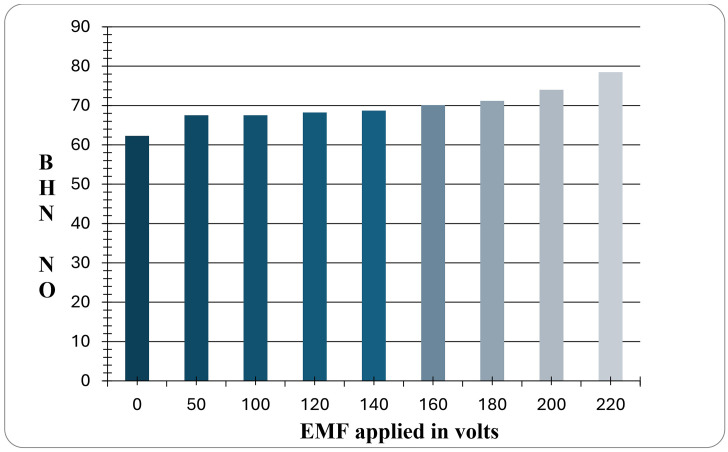
BHN values for different EMF.

Brinell Hardness measurements were initially carried out on as-received specimens and grain-refined specimens. Hardness measurements were conducted at various locations in each case, and an average of four values from different locations was considered for analysis. Fig 12 shows the variation of hardness values for LM 25 without addition for varying EMF.

A closer look at this graph reveals a rising trend in hardness readings at 180, 200, and 220 volts. The maximum hardness values are observed for the samples exposed to 220V, while the minimum hardness value is observed at 0 volts. BHN is obtained for the specimen that is not treated with any refiner and not subjected to an electromagnetic effect. This indicates that the electromagnetic field has a significant impact on the hardness values of the alloy.

Variation of hardness values for LM 25 with the addition of AL-10%TI refiner for different EMF

**Fig 13:**
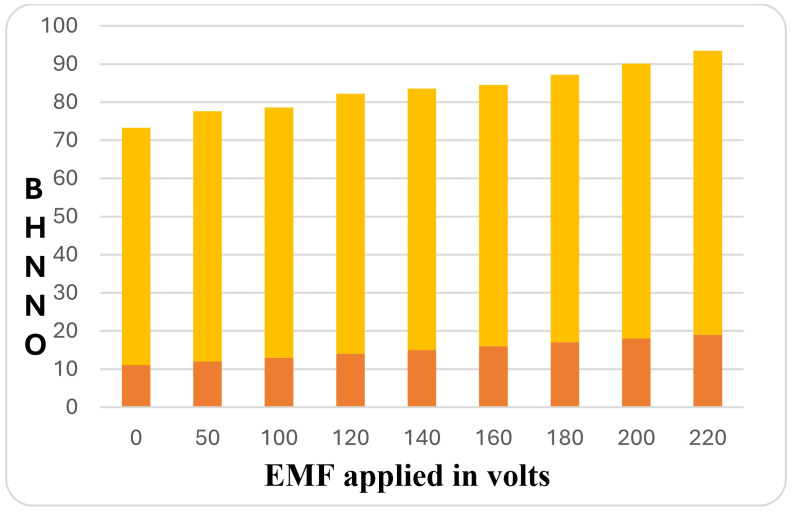
BHN values for various EMF applied in volts for Al-Si7Mg with refiner.

Fig 13 shows the variation of hardness values for different EMF values applied and treated with AL-10%TI refiner, these figure reveal that there is an increase in hardness of material at 180, 200 and 220volts of EMF, further it is also seen that with increase in the EMF values, the hardness values also increases indicating that the electromagnetic induction influences the hardness property of the alloy. The maximum hardness value is observed at 180, 200, and 220 volts, while the minimum values are observed at 0 and 50 volts.

The contrasting trends observed between hardness (monotonic increase) and ultimate tensile strength (non-monotonic for the non-refined alloy) are attributed to the distinct microstructural parameters that govern each property. Hardness, measured via Brinell indentation, is a surface property predominantly controlled by resistance to localized plastic deformation, which is strongly correlated with grain size through the Hall-Petch relationship [46,47]. The combined treatment of electromagnetic agitation and Al-10%Ti refiner produces a progressively finer grain structure with increasing voltage, leading to a consistent, near-linear rise in hardness.

Conversely, ultimate tensile strength is a bulk property sensitive not only to grain size but also to the integrity of the entire microstructure, including the presence of casting defects such as gas porosity, oxide bifilms, and interdendritic shrinkage [34,48,49]. For the alloy without a grain refiner (Fig 9), electromagnetic agitation beyond an optimal intensity (~120 V) generates excessive turbulence. This turbulence increases the entrapment of oxides and gases, introducing defects that act as stress concentrators and crack initiation sites [34]. Thus, while grain refinement continues to increase hardness, the detrimental effect of these internal defects begins to outweigh the Hall-Petch strengthening in tensile loading, resulting in the observed peak and subsequent decline in UTS. In the presence of the Al-10%Ti refiner (Fig 10), the abundant, well-dispersed TiAl₃ nucleants provide a more stable solidification front, mitigating defect formation even at higher EMF voltages [40,50]. This allows both hardness and tensile strength to benefit synergistically from the continuous grain refinement, leading to their concurrent increase up to the maximum applied voltage.

Recent research confirms that subjecting Al-7Si alloys to external energy fields (e.g., electromagnetic, ultrasonic) in conjunction with grain refiners promotes non-dendritic, globular grain structures through mechanisms such as cavitation and acoustic streaming, leading directly to enhanced tensile strength and ductility; a trend consistent with our observations. Notably, a very recent study by Wang et al. [44] specifically demonstrates that ultrasonic treatment coupled with an Al-5Nb-1B master alloy effectively refines both Si phases and α-Al dendrites in an Al-7Si alloy, resulting in superior ultimate tensile strength and elongation, thereby providing direct and contemporary experimental support for the synergistic mechanism observed in our work. These findings align with other studies on ultrasonic processing with Y₂O₃ nanoparticles [51] and broader overviews of non-dendritic microstructure formation [52].

### 3.3 Dry sliding wear evaluation

Dry sliding wear tests were performed using an instrumented Pin-On-Disc machine. Parameters such as load, speed, and test duration were varied. The weight loss and wear rate of specimens under different conditions were determined for each specimen.

#### 3.3.1 Wear rate vs load for AL-Si7Mg without refiners.

**Fig 14:**
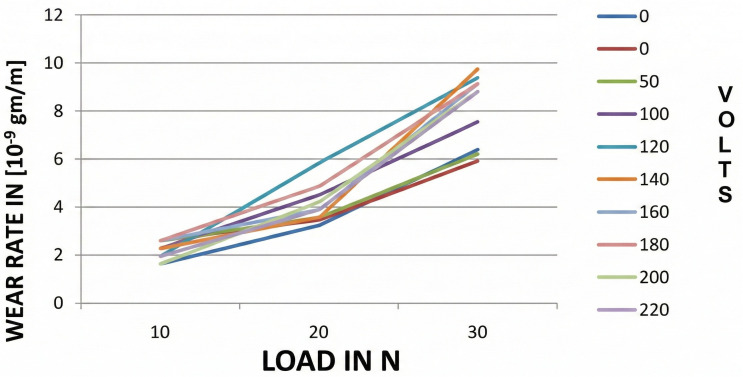
Variation of wear rate versus load for various voltages applied without refiner addition.

Fig 14 shows the variation of wear rate versus load applied for AL-Si7Mg alloy casting subjected to different EMF values without the addition of refiners. The graphic shows how the wear rate rises in tandem with an increase in load. The maximum rate of wear is seen in the specimen, without the addition of refiners and without being subjected to an electromotive force (emf). However, upon subjecting the casting to varying emf, a reduction in wear rate is observed.

#### 3.3.2 Wear rate vs load for AL-Si7Mg with (AL-10%Ti) refiners.

**Fig 15:**
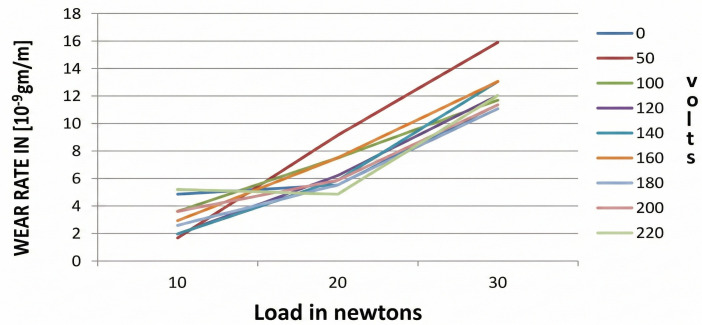
Variation of wear rate versus load for various voltages applied with refiner addition.

Fig 15 shows the wear rate versus load applied for AL-SI7Mg alloy casting subjected to different emf values with the addition of a refiner. It illustrates how the wear rate rises in tandem with an increase in load. The significant improvement in wear resistance observed in samples treated with both Al-10%Ti and EMF (Fig 15) can be attributed to the synergistic microstructural refinement achieved by the combined treatment. Electromagnetic agitation ensures the uniform dispersion of TiAl₃ nucleants from the refiner throughout the melt, promoting the formation of a fine, equiaxed grain structure upon solidification. According to the Hall-Petch relationship, this grain refinement directly enhances the material’s hardness and yield strength [46,47], which are critical parameters for resisting abrasive and adhesive wear mechanisms. A finer grain structure increases the resistance to plastic deformation and subsurface crack initiation during sliding contact.

Furthermore, the forced convection induced by the EMF reduces local solute segregation. It mitigates the formation of casting defects such as microporosity and oxide bifilms [34,46], which often act as nucleation sites for wear debris and accelerate delamination wear. Consequently, the alloy’s ability to withstand higher applied loads without severe material loss is markedly improved. This synergistic effect, where electromagnetic agitation optimizes the refiner’s efficiency to produce a more homogeneous and defect-resistant microstructure, explains the progressive reduction in wear rate with increasing EMF voltage, as clearly evidenced in Fig 15. The maximum weight loss is observed in specimens that are not subjected to an electric field (EMF). However, upon subjecting the casting to varying emf, a reduction in weight loss is observed [48].

The wear test results, which are shown in Figs 14 and 15, reveal that there is a significant resistance to wear in the samples that are grain refined with the addition of AL-10%Ti and are cast under the effect of an electric field (EMF). This shows that improvement in the wear rate is a result of the electromagnetic effect because earlier research shows that only a small extent of wear rate was improved with the addition of a refiner [53].

## 4. Conclusions

- The combined use of Al-10%Ti grain refiner and electromagnetic agitation (EMF) during solidification produces a synergistic effect, significantly refining the microstructure of Al-Si7Mg alloy from a coarse, acicular structure to a fine, closed-grain equiaxed one.
- This microstructural refinement directly enhances key mechanical properties. There is a 15% increase in ultimate tensile strength (to 173.3 N/mm²), a 60% improvement in elongation, and a 21% rise in hardness, with the optimal result achieved at 180V EMF.
- The wear resistance of the alloy is also markedly improved by the combined treatment, demonstrating its comprehensive benefit for enhancing performance.
- Future studies should evaluate the long-term stability, industrial scalability, and specific processing parameters of this combined approach to ensure its practical application.

## Supporting information

S1 File (DOCX)
