# Supplementary material for: Synergistic enhancement of Al-Si7Mg alloy: Strengthening mechanical properties through combined electromagnetic agitation and AL-10%Ti refinement
Source: PLoS One. 2026 Jan 29;21(1):e0341127. doi: 10.1371/journal.pone.0341127 (PMC12854481; doi:10.1371/journal.pone.0341127)
Supplement: S1 File — (DOCX) [file pone.0341127.s001.docx]

**Supplementary Data**

*****************************

**Synergistic Enhancement of Al-Si7Mg Alloy: Strengthening Mechanical Properties through Combined Electromagnetic Agitation and AL-10%Ti Refinement**

Table 1- Chemical composition of Al-Si7Mg (LM25) alloy

| Element | Cu | Mg | Si | Fe | Mn | Ni | Zn | Pb | Sn | Ti | Al |
| --- | --- | --- | --- | --- | --- | --- | --- | --- | --- | --- | --- |
| Max | 0.2 | 0.6 | 7.5 | 0.5 | 0.3 | 0.1 | 0.1 | 0.1 | 0.05 | 0.2 | Bal |

Table 2: Specifications of coil assembly

| Metal used | copper |
| --- | --- |
| No of turns | 1500 |
| Wire gauge | 14 gauge |
| Shape | circular |
| Height | 135 mm |
| Internal diameter | 65 mm |
| External diameter | 80 mm |

Table 3: Details of samples cast with grain refiners and varying (EMF) to the melt

| **Sample No.** | **Particulars EMF applied for 3min** | **Volts** |
| --- | --- | --- |
| 1 | Without refiner, without EMF(As cast) | 0 |
| 2 | Without refiner, without EMF after degasser | 0 |
| 3 | Without refiners, with (EMF) | 50 |
| 4 | Without refiners, with (EMF) | 100 |
| 5 | Without refiners, with (EMF) | 120 |
| 6 | Without refiners, with (EMF) | 140 |
| 7 | Without refiners, with (EMF) | 160 |
| 8 | Without refiners, with (EMF) | 180 |
| 9 | Without refiners, with (EMF) | 200 |
| 10 | Without refiners, with (EMF) | 220 |
| 11 | With refiner, without EMF | 0 |
| 12 | With refiners and magnetic field (EMF) | 50 |
| 13 | With refiners and magnetic field (EMF) | 100 |
| 14 | With refiners and magnetic field (EMF) | 120 |
| 15 | With refiners and magnetic field (EMF) | 140 |
| 16 | With refiners and magnetic field (EMF) | 160 |
| 17 | With refiners and magnetic field (EMF) | 180 |
| 18 | With refiners and magnetic field (EMF) | 200 |
| 19 | With refiners and magnetic field (EMF) | 220 |
